# Clinical Classification of the Diabetic Foot Syndrome Adapted to ICD-10 as a Solution to the Problem of Diagnostics, Statistics and Standardisation

**DOI:** 10.3390/medicina57080817

**Published:** 2021-08-11

**Authors:** Pavel Lukin, Alex G. Kuchumov, Mikhail F. Zarivchatskiy, Tatyana Kravtsova

**Affiliations:** 1 Department of Faculty Surgery #2, Perm State Medical University, 614990 Perm, Russia; vrach400@rambler.ru (P.L.); targs2@gmail.com (M.F.Z.); 2 Department of Computational Mathematics, Mechanics, and Biomechanics, Perm National Research Polytechnic University, 614990 Perm, Russia; 3 Department of Polyclinic Therapy, Perm State Medical University, 614990 Perm, Russia; kravtsova@mail.ru

**Keywords:** diabetes mellitus, diabetic foot disease, disease classification

## Abstract

*Background and Objectives*: To propose a new classification of diabetic foot syndrome adapted for inclusion in the ICD-10 (the ICD-10 is the 10th revision of the International Statistical Classification of Diseases) and providing more reliable data on the number of clinical cases. *Materials and Methods*: A randomized controlled trial was performed. A total of 180 patients (36.6%) discharged from the hospital after surgical treatment and 312 patients (63.4%) who applied independently were observed and analysed. All patients had type 2 diabetes and were comparable in gender, age, duration of diabetes, area and nature of the wound defect. *Results*: We proposed to add the following to the existing ICD-10 and the emerging ICD-11 codes: Edf10.0—insulin-dependent diabetes mellitus with diabetic foot syndrome and Edf11.0—non-insulin-dependent diabetes mellitus with diabetic foot syndrome, where “df” is an acronym for diabetic foot. The new classification designates the seven most frequent areas of the lesion and five degrees of depth of soft tissue lesions. *Conclusions*: The proposed classification adapted for ICD-10 will enable the standardisation of diagnosis, providing a complete picture of this complication of diabetes mellitus, determining the number of amputations and their validity. Accurate statistics will allow for objective funding and timely preventive measures.

## 1. Introduction

Diabetes mellitus is a serious threat to human health around the world [1,2,3]. Diabetes mellitus does not depend on social status, financial status or nationality. According to the latest data published in the *Diabetes Atlas of the International Diabetes Federation (IDF)* (ninth edition), 463 million adults around the world are now living with diabetes [4].

Diabetic foot syndrome combines pathological changes in the peripheral nervous system, arterial and microcirculatory bed and osteoarticular apparatus of the foot, which pose an immediate threat, or the development of ulcerative-necrotic processes and gangrene of the foot [5,6,7,8]. Diabetic foot syndrome occurs in 25% of diabetes mellitus cases, with 85% leading to amputation [9].

To predict and choose treatment approaches, many classifications of diabetic foot syndrome have been proposed, but all have certain drawbacks. Most classifications focus on the visual presentation of lesions in diabetic foot syndrome. For example, the Wagner classification estimates the depth of a trophic ulcer without concomitant lesions such as angiopathy and neuropathy [10]. The University of Texas staging system for diabetic foot ulcers [11], in addition to depth of lesion, assesses the degree of infection and severity of ischemia without considering neurological disorders. The depth of the wound, severity of the infectious process and state of the peripheral blood supply are assessed by the WIFI classification [12,13].

The WIFI (Wound, Ischemia, and Foot Infection) classification is universal and is offered both for patients with and without diabetes mellitus. It contains the key limb status elements needed to gauge the severity of limb threat, which enables physicians to predict amputation risk more accurately. It also enables physicians to compare progress between patient groups undergoing different therapies, thereby improving treatment over time. The Ischemia category of the WIFI classification system is focused on measuring the haemodynamics/perfusion of the patient, using several different diagnostic measurements. Recommended tests include ABI, toe pressure (TP), tcpO2, SPP, and PVR. Of special note is that TP measurements are recommended in all diabetic patients and those with elevated ankle pressures (ABI > 1.3) due to vessel calcification. The WIFI classification allows doctors to predict the risks of developing trophic complications and choose treatment: conservative therapy or surgical intervention [14]. However, when it is applied, it is impossible to assess blood flow in the great vessels and the degree of neuropathy.

The PEDIS classification provides more detailed information about the existing foot lesion at different stages of treatment of a patient with diabetic foot syndrome, considering neurological changes [15]. Although this classification is convenient for scientific research, it is of little use in clinical practice.

More comprehensive reviews of known classifications (e.g., Rutherford classification [16,17], Fontaine classification [18], SAD system [19], etc.) can be found in studies by Mills et al. [12] and Hardman et al. [20].

Funding for the system is based on reliable statistics on the number of cases of diabetic foot syndrome. Contemporary therapeutic approaches and the development of new methods of treating diabetic foot syndrome require certain financial costs [21]. The IDF estimates that the annual healthcare cost of diabetes around the world is USD 760 billion. The costs of treating complications account for over 50% of the direct healthcare costs associated with diabetes [22]. The cost of care for people with diabetes and foot ulcers is 5.4 times higher in the year of the first episode and 2.6 times higher in the year of the second episode [4]. These direct expenses are projected to reach USD 825 billion by 2030 and USD 845 billion by 2045 [1,2,4]. The proposed classifications cannot be applied for statistical accounting and forecasting of economic costs for the prevention and treatment of diabetic foot syndrome.

For the coding of diagnoses and statistics, the ICD-10, adopted in 1990 in Geneva by the World Health Assembly, is widely used. The ICD-10 does not have specific codes for diabetic foot syndrome.

The ICD-10 codes are too extensive. For example, diabetic foot syndrome can be coded as ‘diabetes mellitus with neurological complications’ (E11.4) and ‘with impaired peripheral circulation’ (E11.5). Diabetic foot syndrome includes all these definitions and, in turn, is a ‘specified complication’ (E11.6). The extended version of the ICD-10, with additional numbers in the codes, is not used by most doctors. Statistics require specific data on the incidence of diabetic foot syndrome, but the current ICD-10 cannot provide them.

The purpose of our study is to develop and implement a classification of diabetic foot syndrome, adapted to the ICD-10, which will allow us to trace the dynamics of the course and the results of therapeutic measures in each patient. The classification will allow statistical data on diabetic foot syndrome to be streamlined without changing treatment approaches.

## 2. Materials and Methods

### 2.1. Data Search

A thorough analysis of statistics on diabetes mellitus and its complications published in IDF atlases since 2000 was carried out. PubMed, Medline and Google Scholar were searched for data reporting the prevalence of diabetic foot syndrome published between January 2000 and December 2019. Data on the prevalence of diabetic foot syndrome in the Russian Federation and the Perm region in particular were also assessed. Modern classifications of diabetic foot syndrome were analysed: Wagner, WIFI, University of Texas, PEDIS, and others. Sections E10–E14 of the ICD-10, devoted to diabetes mellitus and its complications, were analysed in detail.

### 2.2. Data Extraction

All data sources on the prevalence of diabetic foot syndrome were verified. Statistics were extracted from primary sources.

Statistical data were excluded from the study if they did not include sufficient methodological information to evaluate the study, if they were not population-based (e.g., carried out in polyclinics or hospitals), if they reported only the prevalence of diabetic foot syndrome in patients with type 1 diabetes, and if they only included people of a specific age group, for example, over 60 years old.

Classifications of diabetic foot syndrome used in certain regions and not used in worldwide practice were not evaluated.

### 2.3. Characteristics of the Research Site

The study was carried out based on the City Clinical Polyclinic No. 2 of the city of Perm.

To obtain statistical information, we used the resources of the Unified Record Information System of the Perm Region, an electronic system for recording patients’ personal data, which was created in 2011 as a result of a modification of the regional information and analytical medical system.

We analysed the ICD-10 codes, which encrypt trophic disorders on the feet in patients with diabetes mellitus. We carried out a comparative assessment of official statistics on the number of diabetic foot syndrome cases in patients with diabetes mellitus in the Perm Region.

### 2.4. Patients

A randomised controlled trial was conducted. Since 2017, we have observed 180 patients discharged from the hospital after surgical treatment for necrectomies and amputations at different levels and 312 patients who applied independently and were observed on an outpatient basis. According to ultrasound diagnostics data on lower-extremity arteries, all patients had a pronounced lesion of the distal parts of the main arteries. It was impossible to perform revascularising operations. The patients were shown complex treatment aimed at arresting the inflammatory process, removing necrotic tissues and stimulating regeneration. All patients had type 2 diabetes mellitus and were comparable in gender, age, duration of diabetes, area and nature of the wound defect.

Patients with bilateral lesions of the feet and/or multiple ulcerative defects of various localisations were excluded from the study.

Written informed consent was obtained from patients. The study was approved by the Ethics Committee of Perm State Medical University, Perm, Russia (protocol No. 3 on 24 March 2017).

### 2.5. Characteristics of the Classification Developed and Used in the Study of Diabetic Foot Syndrome

We have developed a classification of diabetic foot syndrome that can be introduced into the ICD-10 and the ICD-11 under development.

Codes for diabetic foot syndrome adapted for the ICD-10 are proposed: Edf10.0—insulin-dependent diabetes mellitus with diabetic foot syndrome and Edf11.0—non-insulin-dependent diabetes mellitus with diabetic foot syndrome, where ‘df’ stands for diabetic foot. The acronym ‘df’ can also be used in sections E12–E14 of the ICD-10 with the corresponding definitions.

In the developed classification of diabetic foot syndrome, two digits after the dot were added to the proposed codes Edf10.0 and Edf11.0: the first characterises the affected area (7 areas) and the second refers to the depth (5 levels). Separate codes are allocated for amputations: Edf10.8—amputation stump of the lower limb after non-traumatic amputation in insulin-dependent diabetes mellitus with diabetic foot syndrome, without ulcers, and Edf11.8—amputation stump of the lower limb after non-traumatic amputation in non-insulin-dependent diabetes mellitus with diabetic foot syndrome, without ulcers. The letters of the English alphabet indicate the amputation level: t—toe, f—foot, l—lower leg and th—thigh. The side of amputation and/or ulcer is designated: on the right—r and on the left—l. When a trophic defect heals, the code does not specify the depth of the lesion and its localisation.

### 2.6. Statistical Methods of the Study

Statistical data processing was performed using SPSS statistical software version 22.0. The calculation and construction of diagrams reflecting the dynamics of the studied indicators were carried out with the support of Open Office Apache 4. All digital data were processed via variation statistics using Student’s t-test. All studied quantitative signs of a distribution close to the normal value were presented as M ± m, where M is the arithmetic mean, and m is the standard deviation. Differences were considered statistically significant at *p* < 0.05, *t* > 2.

The receiver operating characteristic (ROC) curve was used to assess the diagnostic and statistical efficiency of the proposed diabetic foot syndrome classification, considering the consequences of false decisions. The ROC curve classification of diabetic foot syndrome was used to determine the significance of the presence of diabetic foot syndrome in a patient’s formulated diagnosis, in which doctors can more accurately describe the diagnosis and predict the outcome of diabetic foot syndrome. In addition, ROC curves were also made using the current ICD-10 classification and compared with the proposed classification for diabetic foot syndrome.

## 3. Results

Analysis of the electronic medical data of patients revealed that diabetic foot syndrome is encrypted as follows: M86.6, L97, L08.8, R02, E11.7, E10.7, and I70.2. A total of 492 patients were examined.

**Figure 1:**
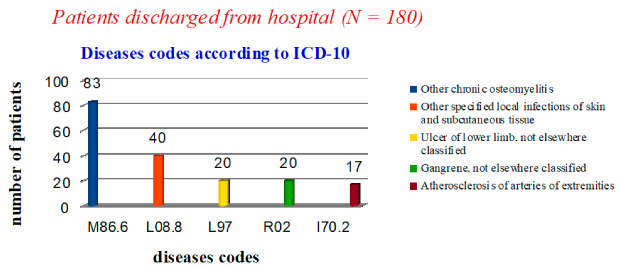
Statistical data on nosologies of patients discharged from the hospital.

Out of 180 patients discharged from the hospital, 110 (61%) underwent disarticulation of the toes. Osteomyelitis (M86.6) was the cause of disarticulation in 50 cases according to ICD-10 codes, in 20 cases trophic ulcer (L97) and in the rest (40 cases) local infections of the skin and subcutaneous fat (L08.8). Seventy patients (39%) underwent local operations (foot amputations according to Lisfranc, Chopard, Sharp), the cause of which in 20 cases was gangrene (R02), in 33 cases osteomyelitis (M86.6) and in 17 cases—atherosclerosis (I70.2). Patients continued to be monitored in the polyclinic until the postoperative wounds were completely healed. Thus, amputations and disarticulations for osteomyelitis of the foot bones officially prevailed in statistical reporting (46%, 83 cases), which was contrary to reality (Figure 1). There were no patients discharged with codes E11.7 and E10.7 reflecting the presence of diabetes mellitus.

**Figure 2:**
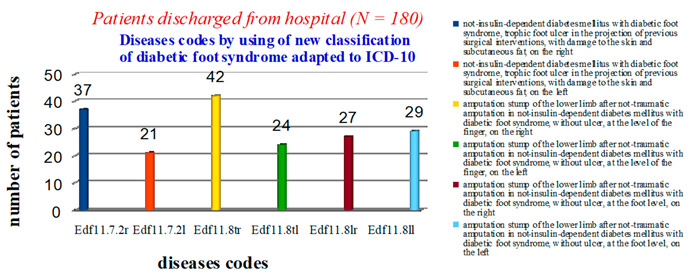
Statistical data on the nosologies of patients discharged from the hospital using a new classification of diabetic foot syndrome, adapted to ICD-10.

Applying our proposed classification (Table 1), we obtained the following results: 58 patients had a diagnosis of Edf11.7.2– non-insulin-dependent diabetes mellitus with diabetic foot syndrome, trophic ulcer of the foot in the projection of previous surgical interventions, with lesions of the skin and subcutaneous adipose tissue (37-r, 21-l), which indicates the discharge of patients with unhealed postoperative wounds; 66 had Edf11.8t—amputation stump of the lower extremity after non-traumatic amputation in non-insulin-dependent diabetes mellitus with diabetic foot syndrome, without ulcers, at the level of the fingers (42-r, 24-l); 56 had Edf11.8l—amputation stump of the lower limb after non-traumatic amputation in non-insulin-dependent diabetes mellitus with diabetic foot syndrome, without ulcers, at the level of the foot (27-r, 29-l) (Figure 2).

**Figure 3:**
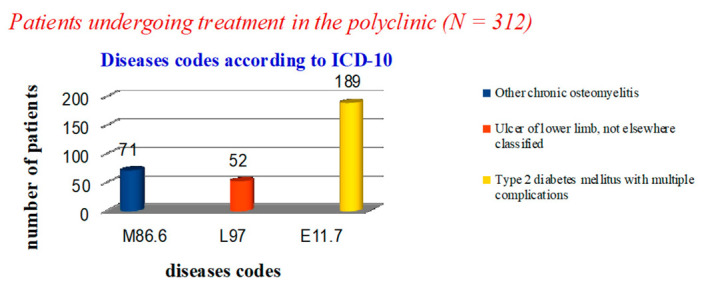
Statistical data on the nosology of patients treated in the polyclinic.

In the polyclinic, only 123 cases in the diagnosis coded by ICD-10 did not contain the phrase ‘diabetes mellitus’: 71 cases—‘osteomyelitis of the toes’ (M86.6) and 52 cases—‘trophic ulcer’ (L97). In 189 patients, the diagnosis was ‘non-insulin-dependent diabetes mellitus with multiple complications’ (E11.7) (Figure 3).

**Figure 4:**
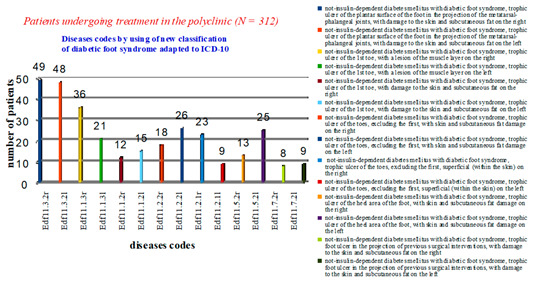
Statistical data on the nosologies of patients treated in a polyclinic using a new classification of diabetic foot syndrome, adapted to ICD-10.

After analysing cases of diabetic foot syndrome in the polyclinic, it was revealed that 97 patients had a diagnosis of Edf11.3.2—non-insulin-dependent diabetes mellitus with diabetic foot syndrome, trophic ulcer of the plantar surface of the foot in the projection of the metatarsophalangeal joints, with lesions of the skin and subcutaneous fat (49-r, 48-l). Eighty-four patients had trophic lesions of the first toe. The diagnosis was coded as Edf11.1. In 57 patients, the depth of the lesion reached the muscle layer (Edf11.1.3), of which 36 had the lesion on the right (r). The rest of the patients (*n* = 131) had other trophic changes in the feet with varying depths of lesion (Figure 4).

Thus, out of 492 patients with diabetes mellitus, 189 (38%) were included in the statistical reporting on complications (the diagnosis code for treatment is E11.7). Extrapolating these data to global statistics, it can be assumed that the official number of cases of diabetic foot syndrome is only a small portion of the real numbers.

To study the reliability of reflecting the presence of diabetic foot syndrome in the diagnosis according to the ICD-10 code, ROC analysis was applied. Our study showed that the official statistics included 38% (189) of patients with diabetic foot syndrome (ICD-10 code E11.7). For comparison, we took from the study subjects an equal number of patients with diabetic foot syndrome (189 people) in whom the ICD-10 diagnosis code did not show the presence of diabetes mellitus and its complications (Table 2 and Table 3).

We assessed the reliability of indicating the presence of diabetic foot syndrome in a patient in his diagnosis according to the ICD-10 code and considered the application of the classification according to the following criteria:

1. Diabetic foot syndrome is absolutely absent;
2. Diabetic foot syndrome is probably absent;
3. Equiprobable presence or absence of diabetic foot syndrome;
4. Probable presence of diabetic foot syndrome;
5. Diabetic foot syndrome is absolutely present.

**Figure 5:**
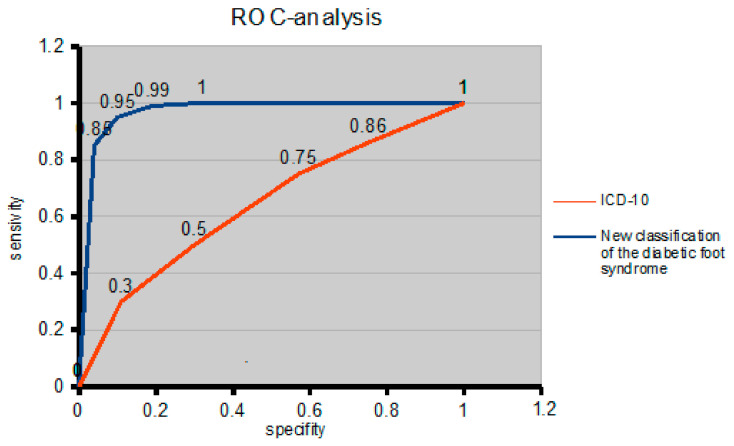
ROC curve to compare the effectiveness of the presence of diabetic foot syndrome in the diagnosis of the ICD-10 and the proposed diabetic foot syndrome classification.

To quantify the application of the classification reflecting the diagnosis of diabetic foot syndrome, a comparative analysis of the areas under the curves was used. The curve of the proposed classification has a larger area than the ICD-10 curve. The area under the curve (AUC) for the new classification of diabetic foot syndrome was 0.96, and the threshold value had a sensitivity of 98% and a specificity of 92%. For comparison, the ROC curve for the ICD-10 had an AUC of 0.58, and the cut-off value had a sensitivity of 62% and a specificity of 58% (Figure 5). Therefore, the proposed classification is more effective in reflecting the presence of diabetic foot syndrome in the diagnosis than the existing ICD-10.

## 4. Discussion

### 4.1. Application and Possibilities of the Proposed Diabetic Foot Syndrome Classification

Diabetes mellitus is a serious threat to the health of people worldwide [23,24,25]. According to the latest statistics published in the *Diabetes Atlas of the International Diabetes Federation (IDF)* (ninth edition) (2019), 463 million adults are currently living with diabetes around the world [4].

One of the reasons leading to the disability of patients is a formidable complication of diabetes mellitus called diabetic foot syndrome, which is an indication for 40%–60% of non-traumatic lower-limb amputations [26,27,28].

According to our data (Table 4), patients with diabetic foot syndrome were predominantly women.

The average age for men who underwent hospital treatment was 64.49 ± 3.90 years, for women 66.27 ± 4.26 years (*p* = 0.911359). The duration of type 2 diabetes mellitus and the presence of a trophic ulcer in men of the main group were noted for 14.54 ± 1.41 and 5.2 ± 1.26 years, respectively, and in women 13.78 ± 1.54 and 5.42 ± 1.6 years (*p* = 0.917471). The area of ulcerative defects in patients of both groups did not differ statistically (*p* = 0.839058).

In polyclinics, the average age for men in the main group was 60.87 ± 3.46 years, for women 61.13 ± 6.08 years (*p* = 0.949506). Type 2 diabetes mellitus duration and trophic ulcers in men of the main group was noted for 11.52 ± 1.42 and 5.44 ± 1.23 years (*p* = 0.857298), respectively, and the presence of trophic defects in men was noted for 5.23 ± 1.12 years and in women 5.6 ± 1.84 years (*p* = 0.942832). The area of ulcerative defects in patients of both groups did not differ statistically (*p* = 0.914011).

Laboratory parameters and stages of trophic ulcer healing are shown in Table 5.

To identify the pathogen, bacterioscopy analysis of smears-prints stained according to Romanovsky–Giemsa was used. Cytological examination of trophic ulcers was carried out. For the reliability of the results of the histological examination, we chose the staining of the preparations with haematoxylin-eosin and according to Van Gieson.

The proposed classification allows us to understand a clear number of trophic defects of the feet of a certain localisation. Table 2 shows the characteristics of trophic defects using the Wagner and Fontaine classifications. These data provide an understanding of the approximate degree of damage and proposed treatment tactics.

The proposed classification of diabetic foot syndrome directly indicates the specific location of the trophic ulcer and the depth of the lesion and is applicable for different types of diabetes. For example, Edf11.1.2r—non-insulin-dependent diabetes mellitus with diabetic foot syndrome, trophic ulcer of the first toe on the right, with lesions of the skin and subcutaneous fatty tissue; Edf11.2.5r—non-insulin-dependent diabetes mellitus with diabetic foot syndrome, trophic ulcer of the toes on the right, excluding the first with osteomyelitis and/or gangrene; Edf10.5.3l—insulin-dependent diabetes mellitus with diabetic foot syndrome, trophic ulcer of the calcaneal region of the foot on the left, with damage to the muscle layer; and Edf11.7.4l—non-insulin-dependent diabetes mellitus with diabetic foot syndrome, trophic ulcer of the left foot in the projection of previous surgical interventions, with damage to bone tissue without osteomyelitis.

The proposed diabetic foot syndrome codes allow for the dynamics of the course of the disease to be traced in a particular patient. Changing the code makes it possible to assess the correctness of the choice of treatment tactics. Changes indicate a worsening of the course of diabetic foot syndrome (Edf11.3.1r—non-insulin-dependent diabetes mellitus with diabetic foot syndrome, trophic ulcer of the plantar surface of the right foot in the projection of the metatarsophalangeal joints, superficial (within the skin), and Edf11.3.2r—non-insulin-dependent diabetes mellitus with diabetic foot syndrome, trophic ulcer of the plantar surface of the right foot in the projection of the metatarsophalangeal joints, with lesions of the skin and subcutaneous adipose tissue). Accordingly, after analysing the treatment performed, one can assume the cause of the deterioration of the wound process.

Epithelisation of the ulcer, according to the proposed classification, is coded with the proposed codes without using the last two digits. For example, if a patient had a trophic defect in the heel region on the left, we used Edf11.5.2l—non-insulin-dependent diabetes mellitus with diabetic foot syndrome, trophic ulcer of the calcaneal region of the foot on the left, with lesions of the skin and subcutaneous fat. When the ulcer heals, the code is changed to Edf11.5l—non-insulin-dependent diabetes mellitus with diabetic foot syndrome, trophic ulcer of the calcaneal region of the foot on the left. Further management of the patient with code Edf11.5l—non-insulin-dependent diabetes mellitus with diabetic foot syndrome, trophic ulcer of the heel of the foot on the left, will always remind doctors that the patient has a history of trophic changes in the feet in a certain area. In the future, this will allow doctors to focus on a specific area when examining the feet to prevent relapse.

For the first time, using the classification, it is possible to trace the dynamics of the course of the wound process after lower-limb amputation at different levels. The proposed codes reflect all levels of amputation: toes, feet, shins, and thighs. In defining the code, we emphasise the non-traumatic nature of the amputation. For example, the amputation stump of the foot in diabetic foot syndrome is coded as Edf11.8lr—amputation stump of the lower limb after non-traumatic amputation in non-insulin-dependent diabetes mellitus with diabetic foot syndrome at the foot level on the right, without ulcers.

In our study, no patients had multiple or bilateral foot lesions. However, in reality, such trophic foot ulcers are quite common and significantly complicate treatment. The new diabetic foot syndrome classification, adapted to the ICD-10, reflects in detail the depth and localisation of such changes. We propose prioritising more serious foot lesions as the main diagnosis. Less severe lesions should be reflected in the accompanying diagnosis. For example, a patient has two trophic ulcers on the plantar surface of the right foot. The first ulcer is in the projection of the first metatarsophalangeal joint with a depth of lesion to the bone. The second is in the projection of the longitudinal arch of the foot, with a depth to the muscle layer. The diagnosis will be as follows: main diagnosis: Edf11.3.5r—non-insulin-dependent diabetes mellitus with diabetic foot syndrome, trophic ulcer of the plantar surface of the right foot in the projection of the metatarsophalangeal joints with osteomyelitis and/or gangrene; concomitant diagnosis: Edf11.4.3r—non-insulin-dependent diabetes mellitus with diabetic foot syndrome, trophic ulcer of the plantar surface in the projection of the arch of the right foot, with damage to the muscle layer.

In the case of bilateral lesions, the main diagnosis indicates the side with the greatest lesion, the concomitant with the least.

Thus, if the classification were applied in a hospital, we could fully track the dynamics of the wound process. The introduction of the classification makes it possible to assess the time interval of the recurrence of trophic disorders. Disease codes reveal the location and depth of the foot lesion. A change in the proposed codes speaks of the effectiveness of treatment, its disadvantages and prognosis for each patient. For the objectivity of performing amputations in a patient, the level can be traced from their history of diagnosis codes.

Globally, this allows practitioners to see how many people in the world have specific foot lesions due to diabetes and analyse the number of patients with a certain level of limb amputation.

The classification is focused on the optimisation and reliability of statistics on diabetic foot syndrome. Validated statistics will determine the level of economic costs for the treatment and prevention of diabetic foot syndrome. For example, it will determine the level of funding for the development of orthopaedic shoes or lower-limb prostheses based on the exact number of patients with affected feet, amputated lower limbs, and disease prognosis.

### 4.2. Comparative Characteristics of Diabetic Foot Syndrome Classifications

Previously proposed classifications of diabetic foot syndrome are diverse and aim at the choice of rational tactics for the surgical treatment of purulent-necrotic lesions of the lower extremities in diabetes, the prognosis of the course of the disease. However, to reflect a specific diagnosis in the record, most of them are voluminous and cannot be adapted to ICD-10.

Today, there are some articles on the comparative characteristics of the noted classifications of diabetic foot syndrome and their application in clinical practice. They are covered in detail in [12,15].

The ICD-10 is widely used for coding diagnoses, adopted in 1990 by the World Health Assembly in Geneva, translated into 43 languages and used in 117 countries.

Diabetes codes are presented in section E, and the most commonly used are E10—insulin-dependent diabetes mellitus, E11—non-insulin-dependent diabetes mellitus and the following quarters:

0—with coma;
1—with ketoacidosis;
2—with kidney damage;
3—with eye lesions;
4—with neurological complications;
5—with impaired peripheral circulation;
6—with other specified complications;
7—with multiple complications;
8—with unspecified complications;
9—no complications.

Unfortunately, most doctors rely on these sections to reflect the diagnosis of diabetic foot syndrome. The main sections of the ICD-10 do not have specific codes for diabetic foot syndrome. All codes are extensive and often incomprehensible. For example, diabetic foot syndrome can be coded as ‘diabetes mellitus with neurological complications’ (E11.4) and ‘with impaired peripheral circulation’ (E11.5) since it includes all the proposed concepts and is more than a ‘specified complication’ (E11.6). The most commonly used codes are E11.7 and E10.7—diabetes ‘with multiple complications’.

At https://icd10cmtool.cdc.gov (accessed on 15 March 2021), the ICD-10 publication is presented with expanded subsections: ‘diabetes with other specified complications’ and ‘diabetes with peripheral circulatory disorders’. In sections E10–E14, additional numbers have been added to the codes. While conducting this research, we faced the fact that only a small number of medical specialists know about these sections of the ICD codes.

The implementation and use of these codes and informational analytical medical computer programs, which contain only the main sections of the ICD-10 for coding diagnoses, make it difficult. This will significantly affect the statistics of diabetic foot syndrome cases and, accordingly, the financing of the industry. The insignificant general and estimated data on the number of diabetic foot syndrome cases worldwide do not allow for an economic assessment of the problem. The IDF provides specific data on the number of patients with diabetes around the world. There are no data on the exact number of diabetic foot syndrome cases and amputations. There are assumptions about the percentage of cases of diabetic foot syndrome relative to the total number of patients with diabetes. Detailed forecasts for the number of patients with diabetes are presented in [1].

Gangrene is often the cause of amputation in diabetic foot syndrome [29,30,31]. Sections E10.5–E14.5 indicate violations associated with microcirculation disorders. Additional numbers determine the presence or absence of gangrene: 1—impaired peripheral circulation without gangrene and 2—impaired peripheral circulation with gangrene.

The causes of gangrene against the background of diabetes is a complex lesion: vascular and severe neurological disorders [32,33]. A thorough examination of patients with diabetes mellitus reveals neuropathy in 70%–100% of cases [34]. Therefore, in our opinion, it is not entirely correct to leave the concept of ‘gangrene’ only in the section on peripheral circulatory disorders.

Trophic changes in the skin of the feet are reflected in sections E10.6–E14.6—diabetes with other specified complications. Subsections contain codes indicating damage to bones and joints. Additional numbers in the codes are 10—neuropathic arthropathy and 18—other diabetic arthropathies. Charcot’s foot is an example of the manifestation of diabetic osteoarthropathy. Often, such a change in the foot leads to the development of serious purulent-necrotic changes. These codes have broad definitions. Charcot’s foot can be attributed to both the first and the second. Many authors consider Charcot’s foot to be a separate complication of diabetes mellitus [35,36]. In our opinion, Charcot’s foot deserves a separate ICD-10 code.

Additional numbers (20, 21, 22 and 28) indicate direct skin changes, such as

- E1x. 620 diabetes mellitus with diabetic dermatitis;
- E1x. 621 diabetes mellitus with foot ulcers;
- E1x. 622 diabetes mellitus with other skin ulcers; and
- E1x. 628 diabetes mellitus with other skin complications.

In these codes, there is no indication of the specific location of the lesion. For example, dermatitis can occur both on the feet and lower legs. If there are manifestations of dermatitis around trophic ulcers, then which code should be used? Additionally, what can be attributed to ‘other skin complications’?

Because of the increase in the number of patients with diabetes, the number of complications is also growing. One of the most serious is diabetic foot syndrome. To understand the exact number of cases of this complication, reliable statistics are needed. The lack of a single code in the ICD-10 for ‘diabetic foot syndrome’ leads to a misunderstanding of the current situation, and all statistical data can be questioned. A correct and understandable diagnosis should be the first priority for the treatment of a patient with diabetic foot syndrome. In many hospitals, the main code is I70.2—‘atherosclerosis of the lower extremities’, and therefore amputation was for atherosclerosis and not for diabetic foot syndrome. There is no unity in the worldwide coding for diabetic foot syndrome. This lack of unity also jeopardises the full funding against diabetic foot syndrome.

The proposed classification of diabetic foot syndrome makes it possible to trace the dynamics of the disease as well as the results of therapeutic measures in each patient. The classification specifies and highlights diabetic foot syndrome as a separate complication of diabetes. Codes allow for an emphasis on the depth and location of the lesion. The use of codes is possible even after trophic ulcer has completely healed. For the first time, codes reflecting the level of amputation with an emphasis on non-traumatic character were included in the classification. We believe that this study’s success in the application of the classification would involve the prioritisation of statistical data on diabetic foot syndrome without changing the treatment approaches.

The disadvantage of the proposed classification is the impossibility of its application in predicting the development of purulent complications; the development of neuropathy and vascular complications.

## 5. Conclusions

The ICD-10, adopted in 1990 in Geneva by the World Health Assembly, in the most recent version and with the latest amendments, requires a certain correction. Modern medicine is becoming a more accurate science and is inextricably linked with mathematical approaches, both in statistics and in the financing of the industry.

Modern classifications of any nosology do not meet the needs of statistics. Any classification directly characterizes the clinical picture of the course of the disease in real time. The proposed classifications of diabetic foot syndrome cannot be combined, since they differ in the criteria for assessing the course of the disease. The classifications are statistically inconvenient and limit the analysis of the full picture of the incidence of diabetic foot syndrome around the world.

The introduction of the ICD-10 codes and the newly presented ICD-11 “diabetic foot syndrome” and the proposed classification of diabetic foot syndrome will allow us to standardize the diagnosis, giving a complete picture of the incidence—as a statistical indicator of this complication of diabetes mellitus. It will determine the number of amputations and their validity.

Our research has shown the expediency of applying the proposed classification. The classification is easy to use and shows the true number of cases of diabetic foot syndrome in daily clinical practice.

The proposed classification of diabetic foot syndrome allows us to trace the dynamics of the course of the disease and the results of therapeutic measures in each patient. The classification is adapted to the ICD-10 and distinguishes diabetic foot syndrome as a separate disease.

The codes allow you to highlight the depth and localization of the lesion. The use of codes is possible even after the complete healing of the trophic ulcer. For the first time, the classification includes codes reflecting the level of amputation with an emphasis on those non-traumatic in nature.

The purpose of creating and applying this classification is to organize statistical data on the number of cases of diabetic foot syndrome and the number of amputations for this reason. The classification is not aimed at changing the tactics of treatment and prognosis of the course of the disease. The classification was created to solve other problems and has an economic significance that cannot be calculated according to modern WIFI and PEDIS classifications.

True statistics will allow us to objectively assess the global situation with an increase in the number of patients with diabetes mellitus and its most serious complication. The exact number of patients with diabetic foot syndrome will determine the funding for the prevention and treatment of diabetic foot syndrome. Timely funding will provide opportunities to prevent amputations of limbs and prevent disability of patients.

## Figures and Tables

**Table 1:** Classification of diabetic foot syndrome adapted to ICD-10.

**Lesion Degree**	**Lesion Area (Edf10.X.x., Edf11.X.x)**	**Lesion Depth (Edf10.x.X, Edf11.x.X)**
Edf10.0 Edf11.0	1—trophic ulcer of the 1st toe; 2—trophic ulcer of the toes, excluding the first; 3—trophic ulcer of the plantar surface of the foot in the projection of the metatarsophalangeal joints; 4—trophic ulcer of the plantar surface in the projection of the arch of the foot; 5—trophic ulcer of the heel area of the foot; 6—trophic ulcer of the dorsum of the foot; 7—trophic ulcer of the foot after previous surgical procedures.	1—superficial (within the skin); 2—with lesions of the skin and subcutaneous fatty tissue; 3—with damage to the muscle layer; 4—with damage to bone tissue without osteomyelitis; 5—with osteomyelitis and/or gangrene.
**Non-traumatic amputation**	**Amputation degree (Edf11.8x, Edf10.8x)**	**Lesion and/or amputation side**
Edf11.8 Edf10.8	t—toe p—foot l—lower leg th—thigh	r—from the right l—from the left

**Table 2:** Evaluation of the effectiveness of ICD-10 to reflect the presence of the diabetic foot syndrome in the patient’s diagnosis.

Current State	Solutions Criteria					Total
	1	2	3	4	5	
Presence of diabetic foot syndrome	26	21	51	37	54	189
Absence of diabetic foot syndrome	47	34	55	32	21	189
Sensitivity	0.3	0.5	0.75	0.86	1	
Specificity	0.11	0.3	0.57	0.75	1	

**Table 3:** Evaluation of the effectiveness of the proposed classification of the diabetic foot syndrome to reflect the presence of the diabetic foot syndrome in the patient’s diagnosis.

Current State	Solutions Criteria					Total
	1	2	3	4	5	
Presence of diabetic foot syndrome	0	2	7	19	161	189
Absence of diabetic foot syndrome	129	24	18	11	7	189
Sensitivity	0.85	0.95	0.99	1	1	
Specificity	0.04	0.1	0.19	0.32	1	

**Table 4:** Characteristics of patients’ groups.

Treatment Location	Sex	Average Age (Years)	Duration of Type 2 Diabetes Mellitus (Years)	Ulcer History (Years)	Ulcer Area (cm^2^)	Disease Severity According to Wagner Classification (Stage)	Chronic Arterial Insufficiency (Stage)
Hospital (*n* = 180)	Male (*n* = 88)	64.49 ± 3.90	14.54 ± 1.41	5.2 ± 1.26	10.36 ± 1.92	2–3	4A
	Female (*n* = 92)	66.27 ± 4.26	13.78 ± 1.54	5.42 ± 1.6	11.24 ± 1.48	2	4A
	p t	0.911359 0.11	0.917471 0.10	0.933050 0.08	0.839058 0.20		
Polyclinic (*n* = 312)	Male (*n* = 117)	60.87 ± 3.46	11.52 ± 1.42	5.4 ± 1.23	11.5 ± 1.68	2	4A
	p	0.949506	0.857298	0.942832	0.914011		
	t	0.06	0.18	0.07	0.11		

**Table 5:** Laboratory parameters and stages of trophic ulcer healing.

Patients Laboratory Indices (Averaged)	Hospital (*n* = 180)	Polyclinic (*n* = 312)
Complete blood count		
Erythrocytes, ×10^12^/L	4.06 ± 1.18	4.56 ± 1.58
Haemoglobin, g/L	118.0 ± 3.74	120.0 ± 4.34
Platelets, ×10^9^/L	363.3 ± 3.93	356.8 ± 4.56
Leukocytes, ×10^9^/L	10.04 ± 1.06	10.26 ± 1.78
Erythrocyte sedimentation rate, mm/hour	32.56 ± 3.42	30.54 ± 5.22
Total protein, g/L	71.5 ± 2.69	74.26 ± 2.18
Total bilirubin, ×10^−3^ mol/L	9.7 ± 1.84	8.67 ± 1.96
Glucose, ×10^−3^ mol/L	8.34 ± 1.21	8.42 ± 1.03
Urea, ×10^−3^ mol/L	13.54 ± 2.35	12.9 ± 2.31
Creatinine, ×10^−3^ mol/L	80.8 ± 3.21	83.4 ± 4.62
Aspartate aminotransferase, u/L	14.28 ± 2.14	14.03 ± 2.23
Alanine aminotransferase, u/L	12.5 ± 1.32	11.7 ± 2.42
Microflora of trophic defects		
*S. aureus*	84	192
*S. haemolyticus*	22	21
*S. epidermidis*	39	52
*S. saprophyticus*	13	12
*Ps. aeruginosa*	14	18
*Streptococcus* spp.	8	14
Stages of healing of trophic ulcers		
Granulations appearance (days)	7.6 ± 1.42	9.2 ± 2.64
Appearance of edge epithelialization (days)	25.7 ± 1.92	27.4 ± 1.28
Healing, full epithelization (days)	56.4 ± 2.52	54.8 ± 2.68

## Data Availability

Not applicable.
